# Three-Dimensional Facial Anthropometric Analysis With and Without Landmark Labelling: Is There a Real Difference?

**DOI:** 10.1097/SCS.0000000000007687

**Published:** 2021-04-15

**Authors:** Daniele Gibelli, Annalisa Cappella, Filippo Bertozzi, Davide Sala, Sara Sitta, Damiano Rosario Tasso, Francesca Tomasi, Claudia Dolci, Chiarella Sforza

**Affiliations:** ∗ LAFAS, Laboratory of Functional Anatomy of the Stomatognathic System, Department of Biomedical Sciences for Health, University of Milan; † U.O. Laboratory of Applied Human Anatomy, Policlinico San Donato, San Donato Milanese; ‡ Department of Biomedical Sciences for Health, University of Milan, Milan, Italy.

**Keywords:** 3D facial assessment, anthropometry, landmark labeling, stereophotogrammetry

## Abstract

**Introduction:**

The actual role of landmarks labeling before three-dimensional (3D) facial acquisition is still debated. In this study, several measurements were compared among textured labeled (TL), unlabeled (NL), and untextured (NTL) 3D facial models.

**Materials and Methods:**

The face of 50 subjects was acquired through stereophotogrammetry. Landmark coordinates were extracted from TL, NL, and NTL facial models, and 33 linear and angular measurements were calculated, together with surface area and volume. Accuracy of measurements among TL, NL, and NTL models was assessed through calculation of relative technical error of measurement (rTEM). The intra- and inter-observer errors for each type of facial model were calculated.

**Results:**

Intra- and inter-observer error of measurements increased passing from textured to NTL and NL 3D models. Average rTEMs between TL models, and NTL and NL models were 4.5 ± 2.6% and 4.7 ± 2.8%, respectively, almost all measurements being classified as “very good” or “good.” Only for orbital height and its inclination, mandibular ramus length, nasal convexity, alar slope angle, and facial divergence, rTEM was classified as “moderate” or “poor.”

**Conclusions:**

Accuracy and precision of measurements decrease when landmarks are not previously labeled; attention must be taken when measurements have a low magnitude or involve landmarks requiring palpation.

In the last decades, facial assessment through surface three-dimensional (3D) image acquisition devices, such as stereophotogrammetry and laser scanning, has gained a growing importance: its applications involve several fields, including maxillofacial and aesthetic surgery,^1^,^2^,^3^,^4^,^5^ orthodontics,^6^,^7^,^8^ and clinical genetics.^9^,^10^,^11^ With time, the noninvasive 3D technologies of surface acquisition have overcome the conventional measurements based on two-dimensional (2D) images and the traditional caliper-based approach.^12^,^13^ In fact, although the classical direct anthropometric techniques are inexpensive, they are significantly time-consuming and require specific training of operators both in facial landmarks recognition and caliper positioning and reading. Finally, they require patient's complete cooperation.^14^ On the other side, surface image acquisition devices allow operators to perform a 3D facial reconstruction in a millisecond time, so reducing artifacts due to involuntary facial movements, and extending the analysis to groups of subjects who usually show less cooperation during the acquisition (ie, children). In addition, from the computerized model linear and angular measurements can be automatically extracted once appropriate landmarks are defined on the 3D scan.^15^

The recognition of facial landmarks remains a basilar procedure both in traditional direct anthropometry and in computerized analysis of 3D optical scans, and requests a specific knowledge of each reference point and the procedures for its localization.^16^ Landmarks can be labeled on the subject's face before the 3D acquisition or localized on the 3D facial model after its digital reconstruction. Although labeling facial landmarks in direct anthropometry is a common practice,^17^ the importance of this procedure in the assessment of digital facial models is controversial, as some authors usually label landmarks^9^,^10^,^11^,^18^,^19^ before 3D acquisition and some others do not.^20^,^21^ In addition, the actual increase in accuracy and precision of facial assessment, which may derive from this procedure, is not completely acknowledged, although literature suggests that labeling is preferable.^22^

Aynechi et al tested possible differences from 3D models of labeled and unlabeled (NL) faces, in comparison with direct anthropometry^14^: the authors stated an overall high reliability of 3D image acquisition systems in comparison with direct anthropometry, regardless of landmark labeling. In contrast, reliability was lower for measurements involving ears and those soft tissue landmarks that are not on anatomical boundaries (different tissues or local geometrical edges).

The above-mentioned study provided the first step for verifying the possible advantages which may derive from landmark labeling: however, several aspects still need to be analyzed more in-depth. For example, the study by Aynechi et al was performed on 10 subjects and assessed 18 linear distances, and no angular measurements.^14^ In addition, no information about surface areas and volumes is given, although these measurements have been widely analyzed in different fields of research.^1^,^4^,^23^,^24^

Secondly, the comparison between labeled and NL 3D models was performed on 2 different acquisitions, taken within a limited time: as a consequence, results may have been affected also by involuntary facial movements.^25^

This study aims at giving a contribution to the debate around the importance of landmark labeling: possible differences in reliability (accuracy and precision^14^) in assessing 16 linear distances and 17 angles, together with facial surface area and volume, were measured on 3D facial digital models of 50 individuals. Three different sets of facial models (unlabeled [NL] models, textured labeled [TL] models, and the untextured [NTL] bersion of the latter ones) were acquired from the same individual. Results will provide additional information for settling the dispute concerning the real advantages deriving from landmarks labeling.

## MATERIALS AND METHODS

### Sample Recruitment

Fifty volunteers (12 males and 38 females; age between 18 and 82 years; mean age 48.0 ± 19.3 years) were recruited. The subjects should represent the population of adults routinely scanned in laboratory, and both normal individuals and patients were enrolled. Exclusion criteria were severe facial deformations, recent traumatic events involving the facial area, or presence of beard that cannot be acquired by the stereophotogrammetric device. A written informed consent was obtained from all subjects before facial scans; all procedures were not invasive and did not provide any risk. The study follows the guidelines provided by Helsinki Declaration and was approved by the University ethical committee (26.03.14; no 92/14).

### Three-Dimensional Acquisition

The face of each subject was acquired through VECTRA M3, a static stereophotogrammetric device (VECTRA-3D Canfield Scientific, Inc, Fairfield, NJ). Jewellery was previously removed, and hairs pulled back through a band to expose forehead and ears. The volunteers were requested to sit on a stool in front of the 3 cameras of the instrument and to keep a neutral position.

**FIGURE 1:**
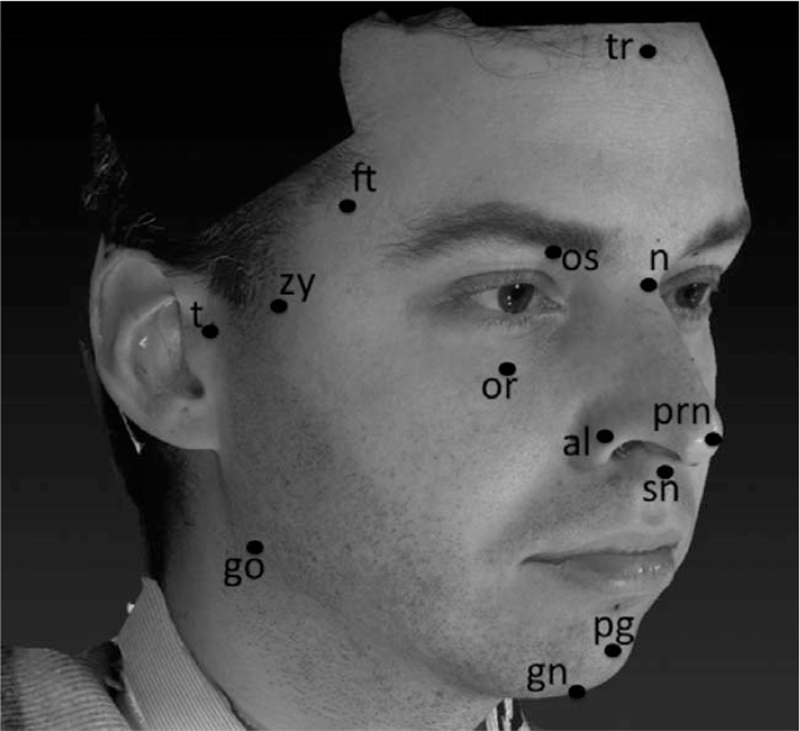
Detail of facial landmarks according to Farkas^16^ labeled on volunteers before the 3D acquisition: tr: trichion; n: nasion; prn: pronasale; sn: subnasale; pg: pogonion; gn: gnathion; t: tragion; ft: frontotemporale; zy: zygion; go: gonion; os: orbitale superius; or: orbitale; al: alare.

A first scan was acquired from each subject without landmark labeling (NL). A series of 20 landmarks was then marked on each face through a black eyeliner, 6 on the midline and 7 on the right and left side (Fig. 1), and a novel facial scan was acquired (textured labeled [TL]). Few seconds passed between the first and the second facial scans.

### Data Elaboration

**FIGURE 2:**
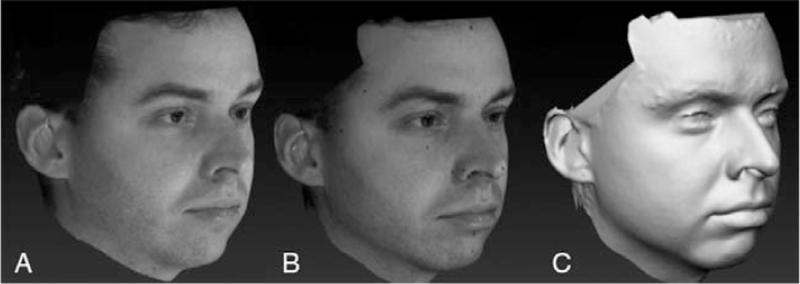
Three different types of 3D facial models: (A) textured unlabeled 3D model (NL); (B) textured 3D model with landmarks labeled through black eyeliner (TL); (C) untextured version of the same labeled 3D model (NTL): colors are automatically removed together with the black dots of labeling.

Each 3D model was elaborated through VAM software (VECTRA Analysis Module, Canfield Scientific, Inc, Fairfield, NJ). The 20 reference points were highlighted on each NL and labelled model through the VAM software which automatically extracted the relevant coordinates. In case of labelled models, the points were pinpointed on the corresponding black marks. Then the labelled models were elaborated again removing the texture (NTL), together with the black dots of landmark labeling. The position of each of the 20 landmarks was again reconstructed without the texture and their coordinates were extracted (Fig. 2).

Coordinates from TL and NTL, and NL 3D models were loaded on Faces software (Department of Biomedical Sciences, University of Milan, Milan, Italy) (specifically developed for the extraction of metrical parameters from coordinates), which automatically calculated 17 linear distances and 16 angles (Supplementary Digital Content, Table 1, http://links.lww.com/SCS/C668).

**FIGURE 3:**
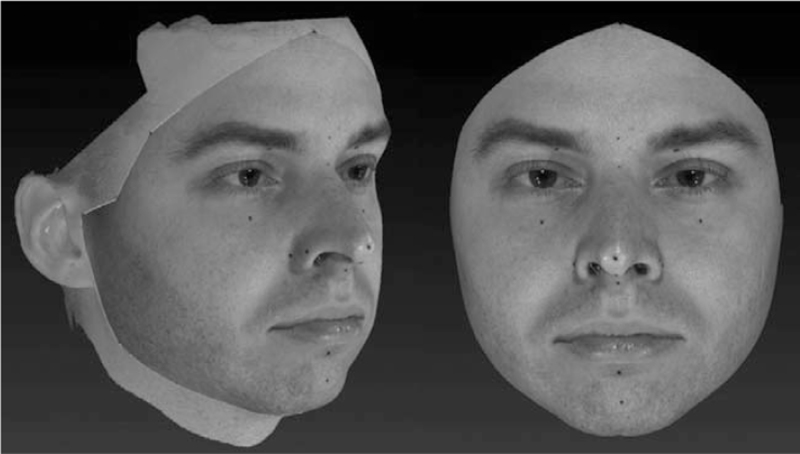
On the left, procedures for definition of FAI (facial area of interest) included between trichion, frontotemporale, zygion, tragion, gonion, gnathion; on the right, automatical removal of external areas.

In addition, a facial area of interest (FAI) was extracted from the 3 sets of 3D models, defined as the facial surface comprised between trichion, frontotemporal, zygion, tragion, gonion, and gnathion facial landmarks (Fig. 3). In case of TL models, the selection of FAI was helped by the presence of eyeliner markers. The entire procedure was found to be well repeatable by literature.^27^ The facial surface area and volume of each FAI were then automatically calculated through VAM software.

### Statistical Analysis

Textured labeled scans were used as reference scans, and the differences with NL and NTL scans were obtained for linear and angular measurements, and FAI surface area and volume, through calculation of relative technical error of measurement (rTEM^28^). One-way ANOVA tests with repeated measures were applied to compare facial dimensions obtained in the 3 facial models. Posthoc tests were made when necessary.

To verify the precision of the measurements and the intra- and inter-observer errors, the same operator and another one repeated the localization of 20 landmarks and extraction of coordinates on 30 randomly extracted facial models in the 3 sets of scans. The respective rTEM values were calculated and evaluated according to the classification provided by Camison et al: <1% excellent; 1%–3.9% very good; 4%–6.9% good; 7%–9.9% moderate; >10% poor.^26^

Paired Student *t* tests were used to verify the significance of TEM values in TL versus NL and TL versus NTL comparisons.

For all comparisons, a *P* value of 0.01 or less was considered significant.

## RESULTS

Intra- and inter-observer rTEM values (precision assessment) are shown in Supplementary Digital Content, Table 2, http://links.lww.com/SCS/C668. For textured 3D models, the performances for all the linear and angular measurements ranged from “good” to “excellent” classifications (with mean rTEMs of 2.1 ± 1.4% and 2.0 ± 1.6%, for intra- and inter-operator assessments, respectively). For NTL and NL 3D models both values increased (3.6 ± 1.8% and 4.6 ± 3.0%, 3.2 ± 2.0% and 3.6 ± 2.5%, respectively), but most of the measurements were included in the category of “good” and “very good” rTEMs. The FAI surface area and volume proved intra- and inter-observer rTEM values at least as “very good” in all the sets of 3D models.

Results of the accuracy assessments between labelled, NL, and NTL models are shown in Supplementary Digital Content, Table 3, http://links.lww.com/SCS/C668. On average, rTEM of all measurements between TL and NTL was 4.5 ± 2.6%, with 30 measurements of 33 classified as “good” at least. Nine linear distances of 17 and 4 angles of 16 showed a statistically significant difference between TL and NTL models (*P* < 0.01), but in 5 cases, including right and left orbital height (os_r_-or_r_), mandibular ramus length (t_m_-go_m_), nasal convexity (sn-n-prn), and facial divergence (t_m_-n - pg–go_m_), the rTEM was classified as “moderate” or “poor.”

The comparison between TL and NL models yielded similar results. In general concordance between measurements slightly decreased in relation to TL/NTL models’ comparison: on average, rTEM was 4.7 ± 2.8%, with 27 measurements of 33 classified at least as “good.” Eight linear distances out of 17 and 9 angles out of 16 showed a statistically significant difference between textured and not textured models (*P* < 0.01), but for left orbital height (os_l_-or_l_), right and left inclination of orbital height (os-or vs TH), nasal convexity (sn-n-prn), and facial divergence (t_m_-n - pg–go_m_), the rTEM was classified as “moderate” or “poor.”

One-ANOVA test verified statistically significant differences in rTEM values between the TL-NTL and NTL-NL, and between TL-NL and NTL-NL comparisons (F: 13.84; *P*: <0.0001). No differences were found between TL-NTL and TL-NL comparisons.

Concordance of FAI surface area and volume between different sets of models was classified as “very good” and “good,” respectively, in all the comparisons.

## DISCUSSION

The introduction of 3D surface acquisition devices has radically changed the field of craniofacial anthropometry and has progressively replaced the role of direct anthropometrical techniques.^29^ Apart from the unvaluable information provided by the inter-landmarks surfaces, the main advantages in comparison with the traditional methods are the reduction of time requested by the acquisition, the limited artifacts due to facial movements, and the chance of extracting surface areas and volumes.^14^

Landmarks labeling before 3D acquisition has become a common practice by several authors^17^,^19^,^27^,^30^; yet, its real impact on the reliability of the subsequent procedures of 3D facial assessment is still incompletely recognized.^22^

Aynechi et al^14^ provided some information concerning this debated topic, analyzing different 3D models acquired through the 3dMDface (3dMD, Atlanta, Georgia, USA) device from the same individuals with and without skin labeling of facial landmarks, and comparing results with those derived from direct anthropometry. The largest differences between labelled and NL models were shown by facial width (zy_r_-zy_l_), middle facial width (t_r_-t_l_), sn-gn distance, and nasal height (n-sn), but in all cases, they were <1 mm. In addition, precision of measurements increased passing from NL to labelled models.^14^

However, the previously cited study provided general information concerning the issue of landmarks labeling, limited to 18 linear distances, whereas no indications are given concerning angles, surface areas, and volumes. In addition, the specific chosen distances may have had a role in influencing the results: in fact, most of the 18 analyzed measurements involved landmarks detectable without previous labeling, as they were in correspondence of anatomical structures (such as endocanthion, exocanthion, cheilion), whereas other landmarks specifically requesting palpation (such as orbitale and orbitale superius) were not included. Finally, the analysis of 2 different acquisitions, respectively for TL and NL 3D models, included involuntary facial movements as a possible source of bias.^14^

The current comparison between TL and NTL models considers the possible influence of preliminary labeling and the presence of a texture in determining the accuracy of facial measurements. On the other side, the comparison between TL and NL models considers possible differences due to landmark labeling and the involuntary facial movements that may have occurred between the 2 consecutive facial scans.

Results showed that precision of all the different types of measurements generally decreased passing from TL to NTL and NL 3D models. However, even without the use of landmarks labelling, most of the measurements were precise, with an rTEM classified at least as “good.”

The most interesting data come from the comparison between measurements assessed on TL and NTL 3D models, differing only in landmark labeling: most of the measurements were classified as accurate at “very good” or “good” degree, with 5 rTEMs ranking as “moderate” or “poor.”. Two of them included the middle point between 2 landmarks, such as the mandibular ramus length (t_m_-go_m_), and facial divergence (t_m_-n – pg-go_m_): in these cases, measurements were obtained using a high number of landmarks, and in detail 4 for the former (t_r_, t_l_, go_r_, go_l_) and 6 for the latter one (t_r_, t_l_, go_r_, go_l_, n, pg). The lower accuracy may be justified by the contribution of different errors in pinpointing the single landmarks. Orbital heights (os-or) showed a “moderate” rTEM as well: in these cases, results can be justified by the need of palpating the superior and inferior orbital edges to adequately detect orbitale and orbitale superius landmarks. In fact, in comparison with the other landmarks whose position can be somehow reconstructed on untextured models with the help of anatomical structures (ie, endocanthion, exocanthion) or the curvature of 3D surface in different planes (ie, nasion, zygion, pogonion), orbitale and orbitale superius are in correspondence of skeletal structures covered by the skin (the supraorbital notch and the middle point of the inferior orbital edge, respectively) and therefore require palpation for their localization. Finally, the “moderate” accuracy of nasal convexity (sn-n-prn) may be explained by the low magnitude of the angle, which may have amplified the error. In fact, literature reports that the smallest the measurements the lowest the repeatability.^31^

The comparison between TL and NL models gave a worse performance than the comparison between TL and NTL models, because of involuntary facial movements. However, this difference was not significant from a practical point of view, as measurements showed a similar distribution within rTEM categories. In addition, average rTEM values were similar in both the comparisons and did not show a statistically significant difference. Moreover, facial measurements with the worst performances were the same, with the addition of orbital height inclinations according to the true horizontal plane (os-or) and alar slope angle (al_r_-prn-al_l_). Both the cases can be justified by the possible influence of subtle movements of eyes and nose between the 2 scans.

Finally, surface area and volume seem to be independent from possible previous labeling procedures, as they yielded a “very good” and “good” rank, respectively, in all the comparisons.

Some limits of the present study need to be discussed: the selected subjects were all adults, in ideal conditions for 3D acquisition. However, the same analysis should be performed in case of not entirely collaborative patients (ie, children).

Another limit concerns the type of measurements: as previously observed, the entity of measurements may influence the results. The present selection of measurements was performed to provide a relatively large number of parameters including distances, angles, surface areas, volumes, and distributed on different planes.

## CONCLUSIONS

In conclusion, the present article provided additional information concerning the long-debated issue around the importance of facial landmarks labeling: the lack of labeled landmarks proved to decrease the precision and the accuracy of all measurements, although they remained within acceptable limits. Special caution should be taken in case of facial landmarks requiring palpation and where the analysis of the curvature of 3D facial surface cannot provide a help for their localization, for measurements with a low magnitude or combined measurements based on more than three landmarks.

## Supplementary Material

SUPPLEMENTARY MATERIAL
