# Supplementary material for: Three-Dimensional Facial Anthropometric Analysis With and Without Landmark Labelling: Is There a Real Difference?
Source: J Craniofac Surg. 2021 Apr 15;33(2):665–8. doi: 10.1097/SCS.0000000000007687 (PMC10292572; doi:10.1097/SCS.0000000000007687)
Supplement: SUPPLEMENTARY MATERIAL [file jcrsu-33-665-s001.docx]

**Supplemental Table 1:** abbreviation and definition for linear and angular measurements: r: right; l: left; m: mean; TH: true horizontal plane

|  | | | **Definition** |
| --- | --- | --- | --- |
| **Linear distances** | **Horizontal** | t_r-_t_l_ | Middle facial width |
|  |  | go_r-_go_l_ | Lower facial width |
|  |  | zy_r_ – zy_l_ | Facial width |
|  |  | al_r_ – al_l_ | Nasal width |
|  | **Vertical** | tr – n | Forehead length |
|  |  | n – sn | Nasal height |
|  |  | sn – pg | Height of the lower face |
|  |  | n – pg | Total facial height |
|  |  | n – prn | Length of the nasal bridge |
|  |  | os_r_ – or_r_ | Right orbital height |
|  |  | os_l_ – or_l_ | Left orbital height |
|  | **Sagittal** | n - t_m_ | Upper facial depth |
|  |  | sn - t_m_ | Midfacial depth |
|  |  | pg - t_m_ | Lower facial depth |
|  |  | pg - go_m_ | Mandibular body length |
|  |  | t_m_ - go_m_ | Mandibular ramus length |
|  |  | prn – sn | Nasal protrusion |
| **Angles** | **Horizontal** | t_r_ - n – t_l_ | Upper facial convexity |
|  |  | t_r_ - prn – t_l_ | Middle facial convexity |
|  |  | t_r_ - pg – t_l_ | Lower facial convexity |
|  |  | go_r_ - pg – go_l_ | Mandibular convexity |
|  |  | t_r_ – go_r_ – pg | Right gonial angle |
|  |  | t_l_ – go_l_ – pg | Left gonial angle |
|  |  | al_r_ - prn – al_l_ | Alar slope angle |
|  | **Frontal** | os_r_ – or_r_ vs TH | Right inclination of the orbital height versus the true horizontal plane |
|  |  | os_l_ – or_l_ vs TH | Left inclination of the orbital height versus the true horizontal plane |
|  | **Sagittal** | n - sn – pg | Facial convexity (excluding nose) |
|  |  | n - prn – pg | Facial convexity (including nose) |
|  |  | sn - n – prn | Nasal convexity |
|  |  | n - prn - sn | Nasal tip angle |
|  |  | (t_m_ - n) - (pg – go_m_) | Facial divergence (midfacial to mandibular plane angle) |
|  |  | os_r_ – or_r_ – t_r_ | Right angle between orbital height and or-t distance |
|  |  | os_l_ – or_l_ – t_l_ | Left angle between orbital height and or-t distance |

**Supplemental Table 2:** rTEM values for measurement precision and intra- and inter-observer error for all values taken on textured labelled (TL), untextured labelled (NTL) and unlabelled 3D models (NL)

|  | | | **TL** | | **NTL** | | **NL** | |
| --- | --- | --- | --- | --- | --- | --- | --- | --- |
|  |  |  | **Intra-operator** | **Inter-operator** | **Intra-operator** | **Inter-operator** | **Intra-operator** | **Inter-operator** |
| **Linear distances** | **Horizontal** | t_r-_t_l_ | 0.6* | 0.6* | 1.1# | 1.0* | 0.4* | 0.7* |
|  |  | go_r-_go_l_ | 1.4# | 0.4* | 1.8# | 3.6# | 3.2# | 3.1# |
|  |  | zy_r_ – zy_l_ | 0.8* | 0.6* | 1.3# | 1.3§ | 1.5# | 1.9§ |
|  |  | al_r_ – al_l_ | 1.1# | 1.4# | 1.6# | 1.1# | 0.8* | 1.0# |
|  | **Vertical** | tr – n | 3.7# | 3.6# | 4.7§ | 4.2# | 4.3§ | 3.9# |
|  |  | n – sn | 0.9* | 3.8# | 4.9§ | 3.3# | 4.1§ | 4.0§ |
|  |  | sn – pg | 1.7# | 1.2# | 4.4§ | 3.7§ | 3.8# | 6.4§ |
|  |  | n – pg | 1.8# | 2.0# | 3.0# | 2.1# | 2.6# | 2.8# |
|  |  | n – prn | 0.6* | 4.3§ | 4.4§ | 3.7# | 4.8§ | 4.4§ |
|  |  | os_r_ – or_r_ | 3.9# | 1.4# | 8.0≈ | 10.1x | 5.6§ | 6.4§ |
|  |  | os_l_ – or_l_ | 4.6§ | 2.4# | 7.7≈ | 13.1x | 5.5§ | 7.8≈ |
|  | **Sagittal** | n - t_m_ | 1.3# | 1.3# | 2.7# | 1.7# | 1.3# | 1.3# |
|  |  | sn - t_m_ | 1.5# | 1.1# | 3.0# | 3.0# | 1.6# | 1.8# |
|  |  | pg - t_m_ | 1.2# | 1.0# | 2.8# | 2.9# | 1.5# | 1.6# |
|  |  | pg - go_m_ | 1.1# | 1.0# | 5.2§ | 7.3≈ | 4.8§ | 3.8# |
|  |  | t_m_ - go_m_ | 3.9# | 2.4# | 2.7# | 4.7§ | 1.6# | 3.0# |
|  |  | prn – sn | 4.5§ | 2.0# | 4.5§ | 8.1≈ | 5.5§ | 8.1≈ |
| **Angles** | **Horizontal** | t_r_ - n – t_l_ | 0.9* | 0.9* | 2.1# | 1.7# | 1.0# | 0.9* |
|  |  | t_r_ - prn – t_l_ | 0.8* | 0.7* | 1.8# | 1.5# | 1.0# | 0.6* |
|  |  | t_r_ - pg – t_l_ | 1.0# | 0.9* | 1.8# | 2.0# | 1.2# | 1.1# |
|  |  | go_r_ - pg – go_l_ | 1.2# | 0.9* | 4.9§ | 5.2§ | 4.5§ | 4.1§ |
|  |  | t_r_ – go_r_ – pg | 2.3# | 1.1# | 4.9§ | 4.4§ | 3.7§ | 4.5§ |
|  |  | t_l_ – go_l_ – pg | 1.8# | 1.5# | 5.1§ | 6.2§ | 3.9# | 4.2§ |
|  |  | al_r_ - prn – al_l_ | 4.1§ | 6.9§ | 4.0§ | 3.9# | 4.1§ | 2.9# |
|  | **Frontal** | os_r_ – or_r_ vs TH | 2.6# | 4.0§ | 5.1§ | 6.3§ | 2.9# | 5.0§ |
|  |  | os_l_ – or_l_ vs TH | 2.9# | 4.1§ | 3.7# | 8.1≈ | 2.6# | 3.6# |
|  | **Sagittal** | n - sn – pg | 0.6* | 0.4* | 2.1# | 1.9# | 0.7* | 2.0# |
|  |  | n - prn – pg | 0.9* | 0.8* | 1.0# | 0.8* | 1.0# | 0.9* |
|  |  | sn - n – prn | 1.8# | 4.9§ | 9.5≈ | 8.9x | 6.4§ | 11.6x |
|  |  | n - prn - sn | 0.6* | 0.8* | 2.6# | 2.5# | 2.4§ | 3.2# |
|  |  | (t_m_ - n) - (pg – go_m_) | 4.6§ | 3.5§ | 5.5§ | 8.8≈ | 9.6≈ | 9.2≈ |
|  |  | os_r_ – or_r_ – t_r_ | 4.4§ | 2.1# | 3.5# | 6.6§ | 3.3# | 3.1# |
|  |  | os_l_ – or_l_ – t_l_ | 4.2§ | 2.8# | 2.6# | 6.9§ | 3.1# | 3.6# |
| **FAI surface area** | | | 0.0* | 0.1* | 0.4* | 0.4* | 0.7* | 1.0# |
| **FAI volume** | | | 0.1* | 0.1* | 1.1# | 1.1# | 1.8# | 1.8# |

All the values are expressed in percentage; r: right; l: left; m: mean; TH: true horizontal plane; FAI: facial area of interest; *: excellent rTEM; #: very good; §: good; ≈: moderate; ×: poor (Camison et al. 26).

**Supplemental Table 3:** Measurement accuracy: p values from 1-w ANOVA test and rTEM, results of Student’s t-test for all the measurements taken on textured (TL) vs. untextured labelled (NTL), and vs. unlabelled (NL) facial models

|  | | | **One-way ANOVA p** | **TL vs. NTL** | | **TL vs. NL** | |
| --- | --- | --- | --- | --- | --- | --- | --- |
|  |  |  |  | **rTEM (%)** | **Student p** | **rTEM (%)** | **Student p** |
| **Linear distances** | **Horizontal** | t_r-_t_l_ | 0.719 | 1.0# | 0.719 | 0.9* | 0.054 |
|  |  | go_r-_go_l_ | 0.001 | 4.0§ | 0.001 | 3.9# | 0.002 |
|  |  | zy_r_ – zy_l_ | 0.005 | 1.5# | 0.005 | 1.5# | 0.000 |
|  |  | al_r_ – al_l_ | 0.002 | 1.9# | 0.002 | 2.5# | 0.001 |
|  | **Vertical** | tr – n | 0.071 | 5.9§ | 0.071 | 5.1§ | 0.373 |
|  |  | n – sn | 0.000 | 4.2§ | 0.000 | 5.1§ | 0.001 |
|  |  | sn – pg | 0.488 | 3.9# | 0.488 | 5.1§ | 0.035 |
|  |  | n – pg | 0.001 | 2.8# | 0.001 | 3.5# | 0.000 |
|  |  | n – prn | 0.000 | 4.8§ | 0.000 | 5.1§ | 0.000 |
|  |  | os_r_ – or_r_ | 0.004 | 7.8≈ | 0.004 | 7.3≈ | 0.791 |
|  |  | os_l_ – or_l_ | 0.975 | 8.9≈ | 0.975 | 10.9× | 0.000 |
|  | **Sagittal** | n - t_m_ | 0.880 | 3.2# | 0.880 | 2.5# | 0.768 |
|  |  | sn - t_m_ | 0.229 | 3.3# | 0.229 | 2.2# | 0.039 |
|  |  | pg - t_m_ | 0.042 | 2.6# | 0.042 | 2.1# | 0.334 |
|  |  | pg - go_m_ | 0.073 | 4.1§ | 0.073 | 4.4§ | 0.008 |
|  |  | t_m_ - go_m_ | 0.003 | 10.9× | 0.003 | 9.5≈ | 0.102 |
|  |  | prn – sn | 0.015 | 6.6§ | 0.015 | 6.8§ | 0.096 |
|  |  |  | **p** | **rTEM (%)** | **p** | **rTEM (%)** | **p** |
| **Angles** | **Horizontal** | t_r_ - n – t_l_ | 0.915 | 2.1# | 0.915 | 1.8# | 0.808 |
|  |  | t_r_ - prn – t_l_ | 0.053 | 2.0# | 0.053 | 1.5# | 0.004 |
|  |  | t_r_ - pg – t_l_ | 0.010 | 1.9# | 0.010 | 1.7# | 0.076 |
|  |  | go_r_ - pg – go_l_ | 0.379 | 4.2§ | 0.379 | 4.8§ | 0.000 |
|  |  | t_r_ – go_r_ – pg | 0.948 | 3.8# | 0.948 | 4.0§ | 0.000 |
|  |  | t_l_ – go_l_ – pg | 0.565 | 4.7§ | 0.565 | 4.8§ | 0.169 |
|  |  | al_r_ - prn – al_l_ | 0.018 | 6.9§ | 0.018 | 7.3≈ | 0.522 |
|  | **Frontal** | os_r_ – or_r_ vs TH | 0.000 | 5.5§ | 0.000 | 7.8≈ | 0.000 |
|  |  | os_l_ – or_l_ vs TH | 0.026 | 6.9§ | 0.026 | 7.3≈ | 0.002 |
|  | **Sagittal** | n - sn – pg | 0.102 | 0.9* | 0.102 | 1.0# | 0.857 |
|  |  | n - prn – pg | 0.000 | 1.2# | 0.000 | 1.4# | 0.000 |
|  |  | sn - n – prn | 0.000 | 8.4≈ | 0.000 | 7.5≈ | 0.000 |
|  |  | n - prn - sn | 0.438 | 2.7# | 0.438 | 3.1# | 0.851 |
|  |  | (t_m_ - n) - (pg – go_m_) | 0.000 | 10.0× | 0.000 | 11.3× | 0.000 |
|  |  | os_r_ – or_r_ – t_r_ | 0.594 | 4.8§ | 0.594 | 5.2§ | 0.005 |
|  |  | os_l_ – or_l_ – t_l_ | 0.097 | 5.9§ | 0.097 | 5.8§ | 0.497 |
|  | | | **p** | **rTEM (%)** | **p** | **rTEM (%)** | p |
| FAI surface area | | | 0.474 | 2.6# | 0.474 | 3.6# | 0.059 |
|  | | | **P** | **rTEM (%)** | **P** | **rTEM (%)** | p |
| FAI volume | | | 0.529 | 4.3§ | 0.529 | 6.2§ | 0.10 |

r: right; l: left; m: mean; TH: true horizontal plane; FAI: facial area of interest; *: excellent rTEM; #: very good; §: good; ≈: moderate ; ×: poor (Camison et al.^26^).
